# Supplementary material for: Global Trends and Correlates of COVID-19 Vaccination Hesitancy: Findings from the iCARE Study
Source: Vaccines (Basel). 2021 Jun 17;9(6):661. doi: 10.3390/vaccines9060661 (PMC8235754; doi:10.3390/vaccines9060661)
Supplement: Supplementary file 1 [file vaccines-09-00661-s001.zip › vaccines-1229425-supplementary.pdf]

Supplementary Table S1. Recruitment numbers across individual countries (N=32,028), and survey periods mapped onto the COVID-19 epidemiology

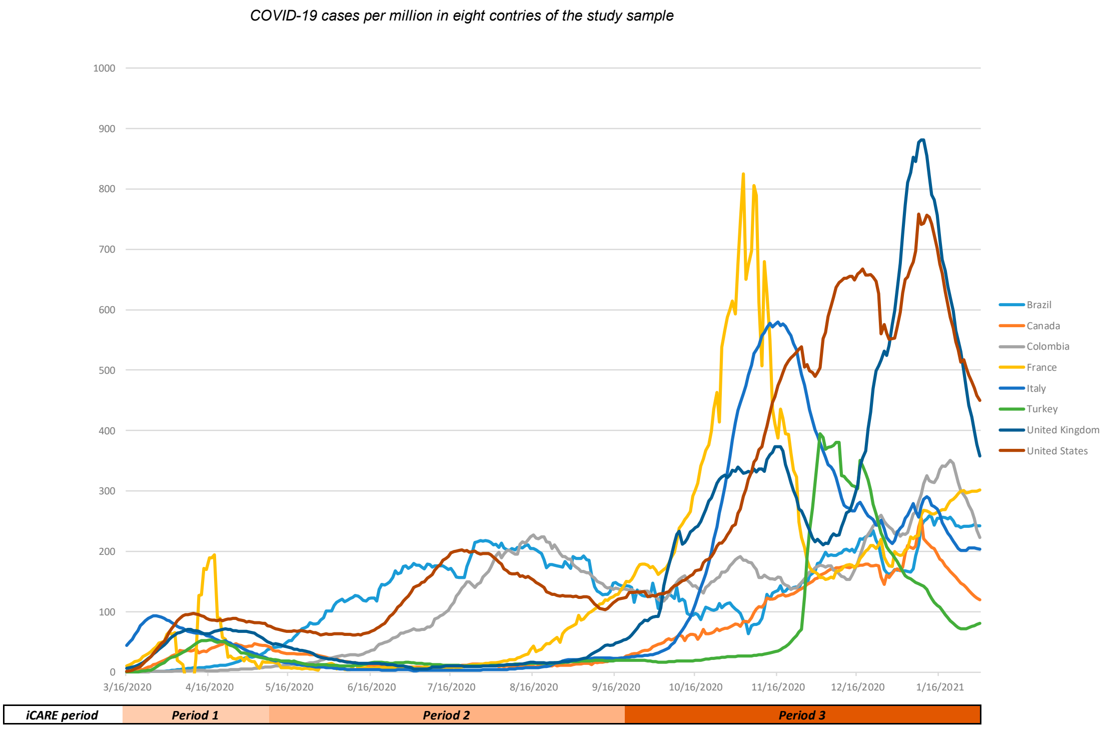

	**period(Period of survey response)**			
	March,April, May	*June, July, August, September (1st half)*	*September (2nd half), October, November, December, January 2021*	*Total*
**Brazil**	1123	920	281	2324
**Canada**	9869	3860	2944	16673
**Colombia**	1265	884	199	2348
**France**	2998	675	290	3963
**Italy**	1436	395	235	2066
**Turkey**	1061	215	151	1427
**UK**	688	162	107	957
**USA**	1491	615	164	2270
**Total**	19931	7726	4371	32028

Supplementary Table S2. STROBE Checklist.

	Item No	Recommendation	Page or manuscript section
**Title and abstract**	1	(*a*) Indicate the study's design with a commonly used term in the title or the abstract	1
		(*b*) Provide in the abstract an informative and balanced summary of what was done and what was found	1
**Introduction**			
Background/rationale	2	Explain the scientific background and rationale for the investigation being reported	2
Objectives	3	State specific objectives, including any prespecified hypotheses	2
**Methods**			
Study design	4	Present key elements of study design early in the paper	2
Setting	5	Describe the setting, locations, and relevant dates, including periods of recruitment, exposure, follow-up, and data collection	2-3
Participants	6	(*a*) Give the eligibility criteria, and the sources and methods of selection of participants	2
Variables	7	Clearly define all outcomes, exposures, predictors, potential confounders, and effect modifiers. Give diagnostic criteria, if applicable	3-4
Data sources/ measurement	8	For each variable of interest, give sources of data and details of methods of assessment (measurement). Describe comparability of assessment methods if there is more than one group	4
Bias	9	Describe any efforts to address potential sources of bias	3-4
Study size	10	Explain how the study size was arrived at	NA
Quantitative variables	11	Explain how quantitative variables were handled in the analyses. If applicable, describe which groupings were chosen and why	3-4
Statistical methods	12	(*a*) Describe all statistical methods, including those used to control for confounding	3-4
		(*b*) Describe any methods used to examine subgroups and interactions	3-4
		(*c*) Explain how missing data were addressed	3
		(*d*) If applicable, describe analytical methods taking account of sampling strategy	NA
		(*e*) Describe any sensitivity analyses	NA
**Results**			
Participants	13*	(a) Report numbers of individuals at each stage of study—eg numbers potentially eligible, examined for eligibility, confirmed eligible, included in the study, completing follow-up, and analysed	NA
		(b) Give reasons for non-participation at each stage	NA
		(c) Consider use of a flow diagram	NA
Descriptive data	14*	(a) Give characteristics of study participants (eg demographic, clinical, social) and information on exposures and potential confounders	4-5
		(b) Indicate number of participants with missing data for each variable of interest	4

			And Supplementary tables
Outcome data	15*	Report numbers of outcome events or summary measures	4-6
Main results	16	(*a*) Give unadjusted estimates and, if applicable, confounder-adjusted estimates and their precision (eg, 95% confidence interval). Make clear which confounders were adjusted for and why they were included	4-8 (Tables 2 and 3)
		(*b*) Report category boundaries when continuous variables were categorized	4-8
		(*c*) If relevant, consider translating estimates of relative risk into absolute risk for a meaningful time period	NA
Other analyses	17	Report other analyses done—eg analyses of subgroups and interactions, and sensitivity analyses	NA
**Discussion**			
Key results	18	Summarise key results with reference to study objectives	8
Limitations	19	Discuss limitations of the study, taking into account sources of potential bias or imprecision. Discuss both direction and magnitude of any potential bias	10
Interpretation	20	Give a cautious overall interpretation of results considering objectives, limitations, multiplicity of analyses, results from similar studies, and other relevant evidence	8-10
Generalisability	21	Discuss the generalisability (external validity) of the study results	10
**Other information**			
Funding	22	Give the source of funding and the role of the funders for the present study and, if applicable, for the original study on which the present article is based	11

Supplementary Figure S1. Vaccine intentions in the overall sample (presenting percentages of individuals that are *Extremely likely, Somewhat likely, Unlikely, and Very unlikely* to get the vaccine)

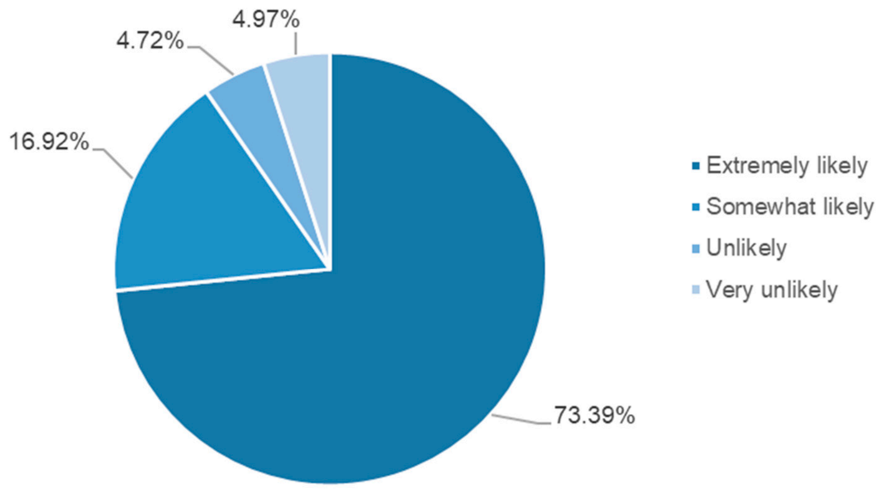

Supplementary Table S3. Principal component analysis of 11 concerns-related items in iCARE (overview of item correlations, component mean values, loadings and graphical presentation of Eigen values (Scree plot))

List of variables used in principal component analysis

**Variable name**	**Description**
concernr_01	*[being infected myself]*
concernr_02	*[the impact of being infected on my health, including dying]*
concernr_04	*[losing my job / family income]*
concernr_06	*[not having enough money for food and/or rent]*
concernr_07	*[infecting other people I live with]*
concernr_08	*[a person with whom I live with being infected]*
concernr_09	*[a family member with whom I do not share my home being infected]*
concernr_011	*[infecting other people in the community]*
concernr_012	*[there not being enough food left on shelves for people to eat]*
concernr_013	*[my country going into an economic recession/depression]*
concernr_014	*[how long it will take for things to go back to normal]*

Correlation matrix for 11 concerns-related variables

	**concernr_01**	**concernr_02**	**concernr_04**	**concernr_06**	**concernr_07**	**concernr_08**	**concernr_09**	**concernr_011**	**concernr_012**	**concernr_013**	**concernr_014**
**concernr_01**	1	0.81581	0.27442	0.28647	0.55862	0.58833	0.47119	0.46131	0.37172	0.18825	0.19095
**concernr_02**	0.81581	1	0.2892	0.31697	0.51823	0.5847	0.46927	0.42347	0.37246	0.17954	0.16269
**concernr_04**	0.27442	0.2892	1	0.84422	0.3308	0.34864	0.29549	0.30363	0.48367	0.41691	0.31698
**concernr_06**	0.28647	0.31697	0.84422	1	0.38657	0.38892	0.31215	0.34207	0.56074	0.39127	0.28122
**concernr_07**	0.55862	0.51823	0.3308	0.38657	1	0.82759	0.62037	0.72947	0.37113	0.19735	0.17081
**concernr_08**	0.58833	0.5847	0.34864	0.38892	0.82759	1	0.70876	0.65841	0.41529	0.23274	0.19936
**concernr_09**	0.47119	0.46927	0.29549	0.31215	0.62037	0.70876	1	0.6694	0.34979	0.20573	0.20952
**concernr_011**	0.46131	0.42347	0.30363	0.34207	0.72947	0.65841	0.6694	1	0.37412	0.22643	0.18332
**concernr_012**	0.37172	0.37246	0.48367	0.56074	0.37113	0.41529	0.34979	0.37412	1	0.49852	0.34282
**concernr_013**	0.18825	0.17954	0.41691	0.39127	0.19735	0.23274	0.20573	0.22643	0.49852	1	0.61225
**concernr_014**	0.19095	0.16269	0.31698	0.28122	0.17081	0.19936	0.20952	0.18332	0.34282	0.61225	1

Component loadings

Rotated Pattern					
		Components			
Components	***Individual variables***	***1***	***2***	***3***	***4***
Health concerns (others)	***[infecting other people in the community]***	0.8557	0.1561	0.1231	0.1075
	[infecting other people I live with].	0.8317	0.1944	0.2830	0.0449
	[a family member with whom I do not share my home being infected]	0.8142	0.1140	0.1937	0.1271
	[a person with whom I live with being infected]	0.8045	0.1954	0.3580	0.0893
Personal financial concerns	***[not having enough money for food and/or rent]***	0.2033	0.9166	0.1052	0.1429
	[losing my job / family income]	0.1579	0.8994	0.0893	0.1802
	[there not being enough food left on shelves for people to eat]	0.2359	0.5418	0.2603	0.4099
Health concerns (self)	***[the impact of being infected on my health, including dying]***	0.3078	0.1575	0.8833	0.0638
	[being infected myself]	0.3465	0.1155	0.8687	0.0960
Social/ economic concerns	***[how long it will take for things to go back to normal]***	0.0938	0.0932	0.0642	0.8861
	[my country going into an economic recession/depression]	0.0930	0.2891	0.0474	0.8386

Mean values of components

Variable	N	Mean	Standard Deviation	Minimum	Maximum
Health concerns (others)	27249	3.30	0.73	1	4
Health concerns (self)	27270	3.00	0.82	1	4
Personal financial concerns	27195	2.28	0.92	1	4
Social/ economic concerns	27258	3.12	0.745	1	4

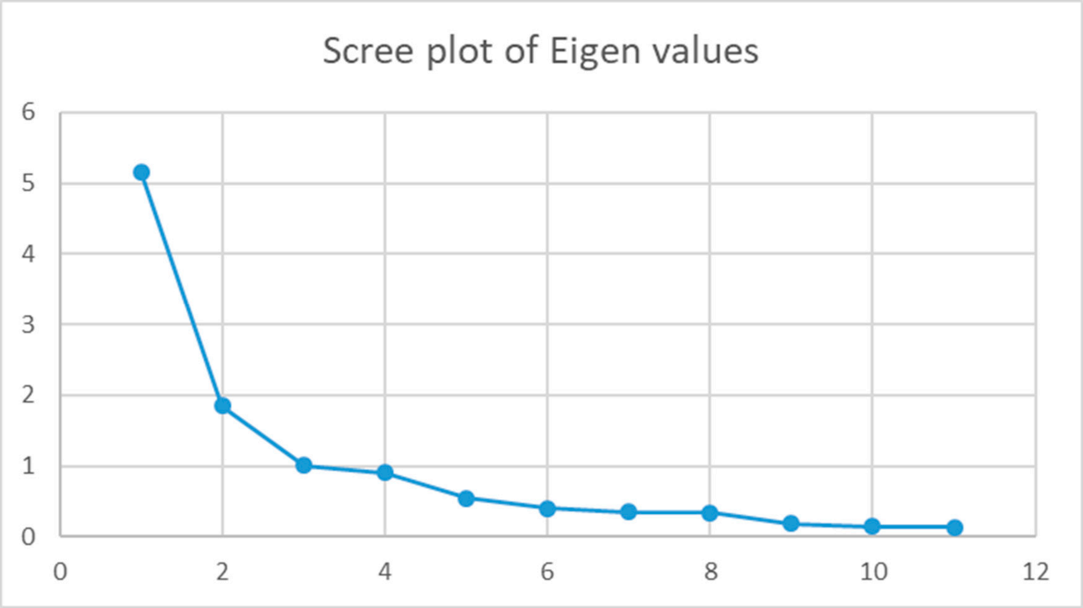

Supplementary Table S4. Socio-demographic characteristics of participants (in the overall sample, and across time).

	**Overall sample**		**Over time**						
			**Period 1**		**Period 2**		**Period 3**		**p-value**
	**N**	**%**	**N**	**%**	**N**	**%**	**N**	**%**	
**Sex**									
Women	22763	72.19	14379	73.2	5309	69.9	3075	71.8	0.0004
Men	8769	27.81	5279	26.9	2282	30.1	1208	28.2	
Missing values	496								
**Age**									
less or equal then 29	8203	26.00	5202	26.4	2223	29.3	778	18.1	<.0001
30-64 years	19130	60.63	12244	62.2	4245	56	2641	61.6	
65 years or more	4219	13.37	2230	11.3	1118	14.7	871	20.3	
Missing values	476								
**Education level**									
High school or lower	6004	22.72	3573	21.3	1576	26.5	855	23.1	<.0001
Graduate or Postgraduate degree	20427	77.28	13217	78.7	4366	73.5	2844	76.9	
Missing values	5597								
**Residential area**									
Rural or Country area	3478	13.21	2442	14.5	678	11.4	358	10.2	<.0001
Suburban or Regional	7151	27.17	4443	26.4	1671	28	1037	29.6	
Urban or City	15695	59.62	9973	59.2	3613	60.6	2109	60.2	
Missing values	5704								
**Perceived average annual household income**									
Bottom third	3207	13.53	2095	13.8	710	13.2	402	12.7	0.1028
Middle third	12812	54.07	8163	53.9	2953	55	1696	53.4	
Top third	7677	32.40	4894	32.3	1703	31.7	1080	34	
Missing values	8332								

Supplementary Table S5. Socio-demographic characteristics of participants (across individual countries).

	Countries															
	Brazil		***Canada***		***Colombia***		***France***		***Italy***		***Turkey***		***UK***		***USA***	
	N	*%*	***N***	*%*	***N***	*%*	***N***	*%*	***N***	*%*	***N***	*%*	***N***	*%*	***N***	*%*
Sex																
Women	1699	73.8	12251	74.8	1617	69.2	2720	69.6	1359	66.4	729	52.1	687	73.4	1701	76.6
Men	603	26.2	4131	25.2	720	30.8	1186	30.4	689	33.6	671	47.9	249	26.6	520	23.4
Missing	22		291		11		57		18		27		21		49	
Age																
less or equal then 29	896	39	3081	18.8	1021	43.8	1020	26	1188	58.6	189	13.5	170	18.3	638	28.8
30-64 years	1319	57.5	10189	62	1258	54	2513	64.1	722	35.6	1162	83.1	647	69.5	1320	59.5
65 years or more	81	3.53	3158	19.2	53	2.27	386	9.85	119	5.86	48	3.43	114	12.2	260	11.7
Missing	28		245		16		44		37		28		26		52	
Education																
High school or lower	419	22.2	3194	23.1	418	22.5	471	13.3	1046	56.7	97	10	97	13.2	262	14.8
Graduate or Postgraduate degree	1469	77.8	10635	76.9	1444	77.6	3062	86.7	800	43.3	872	90	637	86.8	1508	85.2
Missing	436		2844		486		430		220		458		223		500	
Area of living																
Rural or Country area	72	3.79	1524	11.1	109	5.85	828	23.4	571	30.9	22	2.25	195	26.6	157	8.95
Suburban or Regional	82	4.32	4437	32.4	109	5.85	940	26.6	226	12.2	102	10.5	324	44.1	931	53.1
Urban or City	1744	91.9	7753	56.5	1644	88.3	1767	50	1053	56.9	852	87.3	215	29.3	667	38
Missing	426		2959		486		428		216		451		223		515	
Perceived income																
Bottom third	273	14.9	1844	14.8	133	8.13	331	10	213	16.5	83	8.81	88	13.4	242	15.5
Middle third	1350	73.6	6399	51.3	921	56.3	1698	51.4	797	61.5	550	58.4	372	56.7	725	46.5
Top third	211	11.5	4228	33.9	581	35.5	1274	38.6	285	22	309	32.8	196	29.9	593	38
Missing	490		4202		713		660		771		485		301		710	
